# Support and Assessment for Fall Emergency Referrals (SAFER 1) trial protocol. Computerised on-scene decision support for emergency ambulance staff to assess and plan care for older people who have fallen: evaluation of costs and benefits using a pragmatic cluster randomised trial

**DOI:** 10.1186/1471-227X-10-2

**Published:** 2010-01-26

**Authors:** Helen Snooks, Wai-Yee Cheung, Jacqueline Close, Jeremy Dale, Sarah Gaze, Ioan Humphreys, Ronan Lyons, Suzanne Mason, Yasmin Merali, Julie Peconi, Ceri Phillips, Judith Phillips, Stephen Roberts, Ian Russell, Antonio Sánchez, Mushtaq Wani, Bridget Wells, Richard Whitfield

**Affiliations:** 1 Centre for Health Information Research and Evaluation, Swansea University, Swansea UK; 2 Department of Geriatric Medicine at Prince of Wales Hospital, Sydney, Australia; 3 Warwick Medical School, University of Warwick, Coventry, UK; 4 School of Health and Related Research (ScHARR) University of Sheffield, Sheffield, UK; 5 Warwick Business School, University of Warwick, Coventry, UK; 6 Institute for Health Research, School of Health Science, Swansea University; 7 School of Human Sciences, Swansea University, Swansea UK; 8 Department of Geriatric and Stroke Medicine, Morriston Hospital, Swansea, Swansea UK; 9 Prehospital Emergency Research Unit (PERU), Welsh Ambulance Services NHS Trust, Cardiff, UK

## Abstract

**Background:**

Many emergency ambulance calls are for older people who have fallen. As half of them are left at home, a community-based response may often be more appropriate than hospital attendance. The SAFER 1 trial will assess the costs and benefits of a new healthcare technology - hand-held computers with computerised clinical decision support (CCDS) software - to help paramedics decide who needs hospital attendance, and who can be safely left at home with referral to community falls services.

**Methods/Design:**

Pragmatic cluster randomised trial with a qualitative component. We shall allocate 72 paramedics ('clusters') at random between receiving the intervention and a control group delivering care as usual, of whom we expect 60 to complete the trial.

Patients are eligible if they are aged 65 or older, live in the study area but not in residential care, and are attended by a study paramedic following an emergency call for a fall. Seven to 10 days after the index fall we shall offer patients the opportunity to opt out of further follow up. Continuing participants will receive questionnaires after one and 6 months, and we shall monitor their routine clinical data for 6 months. We shall interview 20 of these patients in depth. We shall conduct focus groups or semi-structured interviews with paramedics and other stakeholders.

The primary outcome is the interval to the first subsequent reported fall (or death). We shall analyse this and other measures of outcome, process and cost by 'intention to treat'. We shall analyse qualitative data thematically.

**Discussion:**

Since the SAFER 1 trial received funding in August 2006, implementation has come to terms with ambulance service reorganisation and a new national electronic patient record in England. In response to these hurdles the research team has adapted the research design, including aspects of the intervention, to meet the needs of the ambulance services.

In conclusion this complex emergency care trial will provide rigorous evidence on the clinical and cost effectiveness of CCDS for paramedics in the care of older people who have fallen.

**Trial Registration:**

ISRCTN10538608

## Background

Demand for immediate care through the emergency ambulance service is increasing across the UK and internationally. However up to half of all callers have no clinical need for an emergency department (ED). This includes many older people who have fallen. Though health policy in the UK encourages ambulance services to offer alternative services to such callers, there is little evidence about the safety and effectiveness of new models of care. Alongside training and referral pathways, handheld devices with decision support software could improve the care of this vulnerable patient group.

Falls in older people are recognised internationally as an important issue [1,2], with high human and organisational costs. Reduction in quality of life and physical activity lead to social isolation and functional deterioration, with a high risk of dependency and institutionalisation [3-5]. In the UK, falls account for 3% of total National Health Service (NHS) expenditure [6], and the prevention of falls in older people is a priority [7,8]. Most people who fall do not seek medical advice [9,10] but older people still account for between 12 and 21% of ED visits. Although prevention strategies are effective [8], reduction of falls, injuries and associated morbidity depend on early identification of people at high risk and delivery of interventions across traditional service boundaries [11]. This is reflected in current national and international guidelines [12-14].

In London older people who fall and call 999 for an emergency ambulance response, account for about 60,000 attendances each year or 8% of all emergency ambulance responses [15]. This is similar to the 7.5% of the emergency workload attributable to falls in an urban Emergency Medical Service (EMS) system in the US [16]. Non-conveyance to the ED is high in this group - about 40% in London [15], elsewhere in the UK [17,18] and in the US [16]. Most, (90%), of the falls ambulance staff attend but do not convey to the ED occur in the home [19]. Non-conveyance of patients attended by emergency ambulances is recognised internationally as a safety and litigation risk [20]. Most UK ambulance services have guidelines suggesting that all patients be conveyed to the ED unless the patient refuses to travel to hospital. In practice, however, informal triage by ambulance staff to decide who can be safely left at home has been generally accepted by ambulance services across the UK. However there is no established referral pathway, or requirement to inform, for example, the patient's General Practitioner (GP) about any emergency ambulance call. Little is known about how, in the absence of specific protocols or training to leave older fallers at home, ambulance staff make these decisions. However a US-based study recognised the pragmatic nature of the process of negotiation with the patient about whether to go to hospital [21]. In the UK, qualitative studies have found that crew members deciding whether to take patients to the ED, base decisions on 'intuition' and distance to receiving unit [22-24]. Unfortunately the use of intuition in clinical decision-making is generally considered a source of error and bias [25].

A recent systematic review of the effectiveness of multi-factorial assessment and targeted intervention for falls injury prevention in community and emergency settings concluded that there have been "few large-scale, high-quality randomised trials. Studies are needed that have the power to detect important effects on the number of fall-related injuries and quality of life, so as to resolve uncertainty about the clinical and cost effectiveness" [26] of falls interventions.

This trial addresses an important area of care for older people who fall. It combines a technological innovation with a new model of service delivery across provider boundaries. Evaluation of the costs and benefits of this complex technology will provide valuable information about the development of appropriate care pathways and the potential avoidance of hospital admissions in this vulnerable patient group.

## Methods/Design

### Study Aim

The aim of this research is to assess the costs and benefits of a complex healthcare intervention for older people for whom an emergency ambulance call has been made following a fall. The intervention comprises CCDS software and training for paramedics to help them decide whom to take to hospital and whom to leave at home with referral to a community-based falls service.

### Study Design and Setting

The study is a pragmatic cluster randomised trial with a qualitative component. Allocation will randomise paramedics rather than patients, since the intervention targets health professionals with the aim of studying effects on patient outcomes [27].

### Intervention

The intervention being evaluated is a complex package which comprises paramedic training and CCDS software. The software is installed onto hand-held computers, forming part of an electronic patient record (EPR).

We shall evaluate this package as a whole, in line with the recommendations of the Medical Research Council (MRC) for evaluating complex interventions to improve health [28], as the component parts are interdependent and not easily separated for the purpose of testing.

Paramedics randomly allocated to the intervention group will receive a one-day classroom-based training course. Training will include systematic demonstration of the mechanics and functionality of the software, coupled with practice and supervised role play. Critical reflection and discussion will be encouraged throughout the training. Knowledge reviews will ensure competence and understanding of key aspects of the software functionality. Paramedics will then have a period of four weeks to practise using the new technology. Towards the end of this period, we shall audit their use of the CCDS to ensure they have achieved proficiency.

The CCDS software is on a hand-held tablet Personal Computer (PC), for use by ambulance paramedics attending patients. It will help them to make decisions about the clinical and social care needs of older people who fall. The CCDS software sits alongside the EPR. The CCDS prompts the assessment and examination of injuries associated with the fall, co-morbidity that may have contributed to the fall (e.g. breathlessness or chest pain), psycho-social needs (e.g. cognitive state and ability to undertake activities of daily living) and assessment of environmental risk. Based on these assessments, the CCDS suggests a care plan (e.g. transfer to ED, referral to specific community services and/or patient advice). The clinical assessment component of the CCDS was the intervention in a previous trial with ambulance services [29].

### Control intervention

Patients eligible for inclusion in the trial but attended by control paramedics will receive usual emergency ambulance service care at each study site. This comprises a paper-based decision support system in the form of a structured questionnaire at each site.

### Outcomes

#### Primary

• Interval to the first 999 call or ED attendance categorised as fall; or death

#### Principal

• Interval to the first subsequent 999 call, ED attendance or death (event free period)

• Quality-adjusted event free period

#### Secondary

• Number per patient of further falls for which a 999 call is made

• Number per patient of further 999 calls

• Number per patient of self-reported further falls

• Number per patient of ED attendances

• Number per patient of emergency hospital admissions

• Number per patient of GP (General Practitioner) contacts

• Mortality rate

• Health related quality of life

• Patient satisfaction

• Fall-related self-efficacy (fear of falling)

• Change in place of residence

• Length of hospital stay

• NHS costs

• Personal costs to patient and family

• Pathways of care: proportions of index falls:

◦ conveyed to ED

◦ referred to falls service

◦ referred to GP

◦ left at scene without further care

• Operational indicators: length of time:

◦ spent on scene

◦ in ambulance service job cycle

◦ in episode of care

◦ to respond to 999 call (effect of intervention on response time?)

◦ for falls service to respond

• Quality of care: compliance by paramedics with:

◦ ambulance service treatment protocols

◦ decision support algorithms

◦ clinical documentation

◦ protocol for referral to falls service

These outcomes are consistent with those recommended in recent guidance from the PRevention Of FAlls Network Europe (PROFANE) [30].

### Participants

The trial will be carried out in three ambulance services. In each service we shall recruit paramedics from ambulance stations that serve a General Hospital with a full ED and one or more community-based falls services.

#### Paramedic recruitment and consent

Paramedics are eligible for the trial if they are on active duty at ambulance stations within its catchment area. We shall write to eligible paramedics to invite them to participate. We shall select 24 volunteers from each service at random and allocate half to intervention group and half to controls, again at random. Of these 24 we expect 20 to complete patient recruitment and four to withdraw.

#### Patient recruitment and consent

Patients are eligible for the trial if they are:

• aged 65 or over

• the subject of an emergency ambulance call categorised by the call-taker as a fall without priority symptoms

• attended by a trial paramedic during the recruitment period

• living in the catchment area of a falls service; and

• not living in residential care

To make findings apply to all such patients, we shall not exclude patients with other co-morbidities, including cognitive impairment. However we shall recruit them to the trial only once, namely the first time they meet the inclusion criteria within the study period.

As most emergency callers are distressed and in urgent need, we shall not seek consent by phone or at first attendance. Instead, we shall identify them from routine ambulance service information gathered during the 999 call. Authorised staff from participating services will write to them 7 to 10 days after their falls to tell them about the study and ask them to 'opt out' if they do not wish the trial to contact them again or to access their medical data. They will then give the research team details of patients who do not opt-out for study follow-up.

### Data collection methods

Participating patients will receive questionnaires one and six months after their index fall. Where necessary, we shall administer these through interviews. Questionnaires will measure health-related quality of life through the SF12v2 [31], fear of falling through the Modified Falls Efficacy Scale [32], and self-reported falls. At one month they will estimate patient satisfaction with the Quality of Care Monitor [33]. We shall track patients through the emergency ambulance system, ED departments, GPs and coroners to identify further contacts with these services (or death) within six months. We shall collect diagnostic codes for each contact.

We shall derive time spent on scene (interval between time of arrival of ambulance at patient and leaving the scene of the call), per job cycle (interval between 999 call and completion of call) and per episode (interval between 999 call and completion of care - including time at ED) from routine ambulance and ED records for all calls meeting the study inclusion criteria. We shall assess completeness of clinical documentation relevant to the care of older people who fall from Patient Clinical Records and EPRs completed by paramedics. We shall assess compliance with treatment and referral protocols from ambulance service and falls service records.

In each ambulance service we shall sample 10 older people who fall and are attended by ambulance crews using the new technology. Trial researchers will interview them in depth, using a semi-structured interview schedule to ascertain their views and preferences about the service they received.

We shall also conduct semi-structured interviews or focus groups with intervention group paramedics before and after implementation of the CCDS technology, and with other stakeholders, notably in the falls services. Interview schedules and topic guides will cover: views about the emergency care of older people who fall; the process of decision-making and triage; and issues in implementing the new software. We shall record and transcribe interviews and discussions.

### Follow-up

The research team will work with each participating ambulance service to track patients who meet the inclusion criteria and who have not opted out. They will also liaise with Patient Affairs Managers (or equivalent) at local hospitals and coroners every week to check that these patients have not died. In this way we seek to avoid contacting patients who have recently died.

### Patient involvement

Through two Clinical Research Collaboration Cymru networks - TRUST (Thematic Research network for emergency and UnScheduled Treatment) [34] and Involving People - we have recruited two user representatives to the SAFER 1 Trial. Their role is to attend team meetings and advise on all aspects of the trial, especially where there is contact with patients. In particular they provide feedback on the acceptability of trial questionnaires and patient information. We shall also convene a panel of users to provide more general advice throughout the trial.

### Health economics

We know little about the cost effectiveness of alternative response interventions in emergency ambulance care [35-40]. Therefore economic analysis will estimate the costs of providing the new intervention, the consequences of the scheme for the wider health service (e.g. ED attendances and inpatient admissions) and the costs to patients and families. We shall collect data on the use of health service resources by each patient from paramedic records, GP records, routine hospital records and patient-completed questionnaires. We shall estimate costs by multiplying resource use by unit costs estimated through a micro-costing study within the trial. We shall use the SF6D, derived from the SF12, to estimate the quality-adjusted life years (QALYs) gained from the intervention and economic modelling to estimate the incremental cost-per-QALY. We shall present these ratios with their associated cost-effectiveness acceptability curves. We shall undertake sensitivity analysis to assess the robustness of the results to plausible changes in the configuration of the scheme and other healthcare activity.

### Ethical considerations

The Multi-Centre Research Ethics Committee for Wales has given full ethical approval for the study, including tracking patients across service providers. Although consent mechanisms based on opting out are unusual, two recent studies have received ethical approval to use this mechanism as the only feasible way to include patients in this vulnerable and hard-to-reach group, and thus improve their care [41,42].

To monitor the progress of the trial we have established two independent bodies - Trial Steering Committee (TSC) and Data Monitoring & Ethics Committee (DMEC). The DMEC, with a Clinical Trials Unit Director as chair and members from the fields of geriatrics, public health and statistics together with a user representative, reports to the TSC. The TSC is chaired by a primary care academic and includes members from an ambulance service and emergency medicine, and another user representative.

### Sample size

We designed the trial to detect clinically important changes in the primary outcome - the time to first subsequent reported fall (or death). We judged that we could recruit 20 active paramedics (ten in intervention group, and ten in control group) at each site. As there is no published data on the distribution of time to first reported fall, we estimated the sample size conservatively, using the rate of subsequent falls (or deaths).

From data from participating ambulance services, we expect 250 older people to fall in each site each month. However it will not be possible to identify all who have fallen as eligible for the trial from information given during the emergency call. Furthermore some patients will opt out. Estimating conservatively that we can recruit 133 older people per site per month, a recruitment period of four months will enable us to recruit 500 patients per site, that is 25 per cluster and 1500 in all.

This sample size will yield 80% power when using a 5% significance level to detect a fall in the proportion of participants who make another emergency call for a fall (or death) within six months from 50%, as found in London recently [41], to 40% if, as we expect, the intra-cluster correlation coefficient is less than 0.035. Since this proportion is a binary variable, the time to first reported fall (or death), which is an interval variable, will yield greater power. We shall also have power to detect an effect size of 0.20 (i.e. one fifth of the population standard deviation) in SF12 scores.

### Randomisation and blinding

The 'West Wales Organisation for Randomised Trials in health and social care' (WWORTH) is randomising paramedics between intervention and control. We shall conceal the resulting allocation until we reveal it by inviting individual paramedics to training days. Blinding participants to trial group allocation is neither feasible nor appropriate in a pragmatic trial like this.

Older people who fall and are attended by a control paramedic will receive the participating ambulance service's standard care. As it may not be feasible to blind the dispatchers in ambulance control to the trial group of their paramedics, we shall monitor and, if necessary, manage ambulance dispatch to avoid selection bias, which might manifest itself in a higher transfer or recruitment rate in the intervention group.

### Statistical methods

We shall comply with all standards defined in the CONSORT guidelines [43]. We shall compare measures of process, outcome and cost between intervention and control patients by 'intention to treat'. As we expect many subsequent emergency calls for falls, many participants will call more than once during the trial period. If the intervention is effective, therefore, later attendances by paramedics with the CCDS could dilute the true effect on outcomes. For primary analysis, nevertheless, participants will remain in the group to which they are allocated.

We shall compare our primary and principal outcomes between groups by multi-level survival analysis. This will include separate analyses for later falls (including deaths) and for deaths alone. We shall review all deaths within 72 hours, the typical interval between index fall and referral to falls service. We shall monitor all deaths within the follow-up period of 6 months according to the WWORTH Standard Operating Procedure for Safety Monitoring. We shall compare secondary outcomes between groups using parametric or non-parametric methods as appropriate.

The trial statistician undertaking analyses will be blind to the trial group of all participants. We shall analyse qualitative data thematically using content analysis.

## Discussion

### Strengths

There have been "few large-scale, high-quality randomised controlled trials of the effectiveness of multi-factorial assessment and targeted intervention to prevent falls in community and emergency settings" [26]. Studies are needed that have the power to detect important effects on the number of falls and quality of life, and resolve uncertainty about the clinical and cost effectiveness of falls interventions. This trial responds to this call by evaluating a potentially powerful combination of technological innovation and a new model of service delivery.

### Weaknesses

Since the SAFER 1 trial received funding in August 2006, several issues have delayed implementation, including:

• Radical ambulance service reorganisation took place in England in 2007, with 29 ambulance services reduced through mergers to 12 regional Ambulance Service Trusts.

• Senior staff at each of the participating services changed, including Chief Executive and Director of Information. As a result, the research team has had to renegotiate participation at a time when research was not an organisational priority in England or Wales.

• The national 'Connecting for Health' (CfH) programme [44] introduced the EPR programme into participating ambulance services alongside the SAFER 1 project. Although we explored opportunities for collaborating with CfH EPR software providers, timetables were not compatible and two of the original three ambulance services withdrew from the trial.

Although many ambulance services expressed interest in the SAFER 1 trial, these challenges prevented them from participating. Both of the English ambulance services originally recruited to take part in the study had to withdraw, together with a third English service that was keen to participate.

### Progress

**Figure 1:**
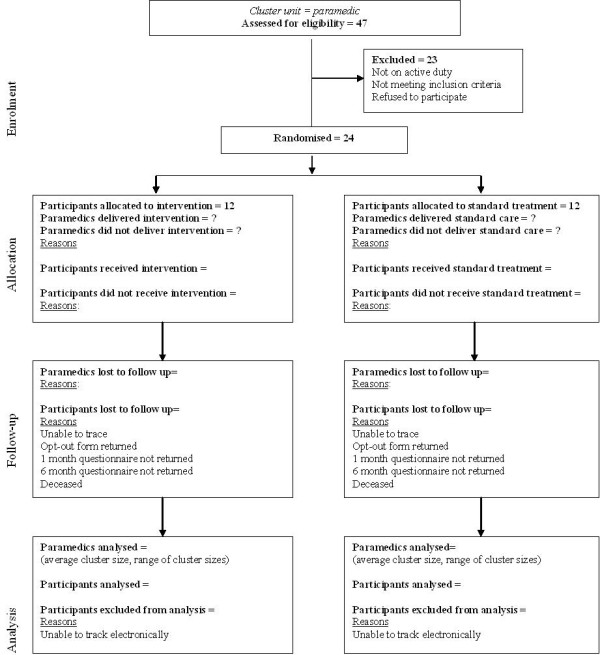
**CONSORT diagram for the South Wales site**.

Fortunately two more English services have recently agreed to participate, and are preparing for the trial. In Wales, where there are no immediate plans to introduce EPR, implementation is underway (Figure 1). Paramedics have been recruited, randomised and trained, the falls pathway has been negotiated, and research governance processes are complete. Study hardware, including computers, docking stations, printers and servers, has been fitted into 13 vehicles in Swansea. We have also negotiated data capture for the trial with security levels acceptable to all parties to the trial in Wales.

## Conclusion

This is a trial of a complex intervention in a challenging setting. Evaluation of this intervention is essential to underpin future purchasing and service development decisions, at both national and local levels. We aim to provide rigorous evidence that will be useful to practitioners, managers and policy makers on the clinical and cost effectiveness of computerised clinical decision support for paramedics caring for older people who have fallen.

## List of abbreviations used

CCDS: (Computerised Clinical Decision Support); CfH: (Connecting for Health); ED: (Emergency Department); EMS: (Emergency Medical Service); EPR: (Electronic Patient Record; GP: (General Practitioner); NHS: (National Health Service); PC: (personal computer); WWORTH: (West Wales Organisation for Rigorous Trials in Health and social care).

## Competing interests

JD is shareholder in, and clinical director of, Plain Healthcare who supply the CCDS software used in the trial. He will play no part in data management or analysis.

## Authors' contributions

HS and JD formulated the research question and conceived the study. All co-authors helped to develop the funded protocol. BW, SG, IH, JP and AS have since refined that protocol. All authors critically reviewed and approved the final manuscript.

## Pre-publication history

The pre-publication history for this paper can be accessed here:

http://www.biomedcentral.com/1471-227X/10/2/prepub
